# Genomic Surveillance of Climate-Amplified Cholera Outbreak, Malawi, 2022–2023

**DOI:** 10.3201/eid3106.240930

**Published:** 2025-06

**Authors:** Lucious Chabuka, Wonderful T. Choga, Carla N. Mavian, Monika Moir, Christian Morgenstern, Houriiyah Tegally, Abhinav Sharma, Eduan Wilkinson, Yeshnee Naidoo, Rhys Inward, Samir Bhatt, G.R. William Wint, Kamran Khan, Isaac I. Bogoch, Moritz U.G. Kraemer, José Lourenço, Cheryl Baxter, Massimiliano Tagliamonte, Marco Salemi, Richard J. Lessells, Collins Mitambo, Ronald Chitatanga, Joseph Bitilinyu-Bango, Mabvuto Chiwaula, Yollamu Chavula, Mphatso Bukhu, Happy Manda, Moses Chitenje, Innocent Malolo, Alex Mwanyongo, Bernard Mvula, Mirrium Nyenje, Tulio de Oliveira, Mathew Kagoli

**Affiliations:** Centre for Epidemic Control and Innovation, School of Data Science and Computational Thinking, Stellenbosch University, Stellenbosch, South Africa (L. Chabuka, W.T. Choga, M. Moir, H. Tegally, E. Wilkinson, Y. Naidoo, C. Baxter, T. de Oliveira); Public Health Institute of Malawi, Lilongwe, Malawi (L. Chabuka, C. Mitambo, R. Chitatanga, J. Bitilinyu- Bango, M. Chiwaula, Y. Chavula, M. Bukhu, H. Manda, M. Chitenje, I. Malolo, A. Mwanyongo, B. Mvula, M. Nyenje, M. Kagoli); University of Florida Emerging Pathogens Institute, Gainesville, Florida, USA (C.N. Mavian, M. Tagliamonte, M. Salemi); Imperial College London, London, UK (C. Morgenstern, S. Bhatt); South African Medical Research Council Centre for Tuberculosis Research, Cape Town, South Africa (A. Sharma); University of Oxford, Oxford, UK (R. Inward, G.R. William Wint, M.U.G. Kraemer); Copenhagen University, Copenhagen, Denmark (S. Bhatt); Environmental Research Group Oxford, Oxford (G.R. WilliamWint); BlueDot, Toronto, Ontario, Canada (K. Khan); University of Toronto, Toronto (K. Khan, I.I. Bogoch); Pandemic Sciences Institute, University of Oxford, Oxford (M.U.G. Kraemer); Biosystems and Integrative Sciences Institute at University of Lisbon, Lisbon, Portugal (J. Lourenço); Kwazulu-Natal Research and Innovation Sequencing Platform, Durban, South Africa (R.J. Lessells, T. de Oliveira)

**Keywords:** cholera, enteric infections, bacteria, genomic surveillance, phylodynamics, climate, flooding, Malawi

## Abstract

In the aftermath of 2 extreme weather events in 2022, Malawi experienced a severe cholera outbreak; 59,325 cases and 1,774 deaths were reported by March 31, 2024. We generated 49 *Vibrio cholerae* full genomes from isolates collected during December 2022–March 2023. Phylogenetic and phylogeographic methods confirmed that the Malawi outbreak strains originated from Pakistan’s 2022 cholera outbreak. That finding aligns with substantial travel between the 2 countries. The estimated most recent ancestor of this lineage was from June–August 2022, coinciding with Pakistan’s floods and cholera surge. Our analysis indicates that major floods in Malawi contributed to the outbreak; reproduction numbers peaked in late December 2022. We conclude that extreme weather events and humanitarian crises in Malawi created conditions conducive to the spread of cholera, and population displacement likely contributed to transmission to susceptible populations in areas relatively unaffected by cholera for more than a decade.

Cholera is an acute diarrheal disease caused by ingestion of food or water contaminated with the bacterium *Vibrio cholerae* (*1*). Since mid-2021, the seventh cholera pandemic, associated with the *V. cholerae* O1 El Tor biotype, has been on an acute upsurge. Several large outbreaks have occurred in endemic and nonendemic countries; countries in Africa were particularly heavily affected (*2*). Those outbreaks have been driven by multiple factors, including extreme weather events, humanitarian crises, overlapping health emergencies (particularly during the COVID-19 pandemic), and consequently overstretched health systems (*3*,*4*).

In Malawi, cholera was first reported in 1973 and has been endemic since 1998. Annual increases in incidence occur during the rainy season (November–May, average temperature 30°C–35°C), particularly in the southern part of the country (*5*). During 2022–2024, Malawi experienced a widespread cholera outbreak that persisted throughout the dry season (May–November, average temperature 13°C); 59,325 cases and 1,774 deaths were reported as of March 31, 2024 (*6*). The outbreak in Malawi unfolded amidst a global surge in cholera outbreaks, intensifying the scarcity of vaccines, tests, and treatments (*7*). Several other countries in southeastern Africa, in particular Mozambique, South Africa, Tanzania, Zambia, and Zimbabwe, were experiencing outbreaks concomitant with the outbreak in Malawi (*7*).

Previous genomic analysis has revealed cholera epidemics in Africa to be associated with transcontinental transmission of *V. cholerae* O1 El Tor sublineages from Asia, followed by regional cross-border spread within Africa (*8*,*9*). To explore the origin and drivers of the 2022–2023 Malawi cholera outbreak, the Public Health Institute of Malawi (PHIM) partnered with the Centre for Epidemic Response and Innovation (CERI), a specialized genomics facility of the Africa Centres for Disease Control and Prevention and World Health Organization Regional Office for Africa (AFRO) (*10*), to perform in-country genomic sequencing of *V. cholerae*. Using phylogenetic and phylogeographic methods alongside epidemiologic modeling that incorporates flooding and vaccination data, we investigated the genomic epidemiology of the cholera outbreak in Malawi, while delving into its climate-amplified implications.

## Methods

### Ethics, Sample Selection, Culture, and DNA Extraction

To investigate the origin of the current cholera outbreak in Malawi, the Public Health Institute of Malawi, supported by CERI and the Climate Amplified Diseases and Epidemics (CLIMADE) program, performed onsite genomic sequencing of local isolates. Isolates were anonymized and did not contain any personal identifiers. The Stellenbosch University Health Research Ethics Committee approved the CLIMADE initiative (BES-2023-24266). The Public Health Institute of Malawi and Ministry of Health National Health Sciences Research Committee approved this study. 

We collected fecal samples from patients with cholera symptoms who sought care in district and central hospitals. We used rapid diagnostic tests (RDTs) to identify the presence of *V. cholerae* and then performed culture on all samples that were RDT positive. We performed drug susceptibility testing on all positive cultures. We sent all positive culture plates to the National Genomic Sequencing Reference Laboratory for DNA extraction; we used the QIAamp DNA Mini Kit 51304 (QIAGEN, https://www.qiagen.com) for extraction and Qubit High Sensitivity DNA kit Q32854 (Thermo Fisher Scientific, https://www.thermofisher.com) on Qubit 4 for quantification. We obtained DNA extracts from 70 *V. cholerae* isolates from samples collected during December 2022–February 2023 from the Southern, Central, and Northern Regions of Malawi. We extracted demographic, clinical, and diagnostic data from the routine cholera surveillance system.

### Sequencing

We prepared libraries using the Illumina DNA library preparation kit and Nextera CD indexes (Illumina, https://www.illumina.com), according to the manufacturer’s protocol. We performed whole-genome sequencing using the NextSeq 1000 instrument with P2 (300) cycle kit reagents (Illumina). We have deposited the genomic sequences into the National Center for Biotechnology Information Sequence Read Archive (BioProject no. PRJNA967700).

### Assembly and High-Quality Single-Nucleotide Polymorphism Calling

We obtained full genomes and single-nucleotide polymorphism (SNP) alignments with our CholeraSeq pipeline (https://github.com/CERI-KRISP/CholeraSeq). In brief, we assessed read quality and trimmed all residual adaptors with fastp (*11*) and used Snippy version 4.6.0 (https://github.com/tseemann/snippy) to perform reference-based assembly against the N16961 strain (GenBank accession nos. NZ_CP028827.1, NZ_CP028828.1) before assembly. We set FreeBayes variant calling thresholds (E. Garrison, G. Marth, unpub. data, https://arxiv.org/abs/1207.3907) as >10× for site coverage, >60 for mapping quality, and >90% for base concordance. We merged individual vcf files using bctfools version 1.15 (*12*). We ran core genome alignment through fastBaps version 1.0.8 (*13*) before recombination screening with Gubbins version 3.2.1 (*14*). We manipulated FASTA files with seqkit version 2.0.0 (*15*) and the Biostrings R package version 2.58 (https://bioconductor.org/packages/release/bioc/html/Biostrings.html). We extracted parsimony informative sites from consensus genome alignments in MEGAX version 10.0.3 (*16*). We downloaded all available *V. cholerae* whole-genome sequencing experiments from the National Center for Biotechnology Information Short Read Archive and the European Nucleotide Archive. We assembled paired-end reads and called SNPs using the same methodology applied to the newly sequenced Malawi strains.

### Phylogenetic Inference with Worldwide Cholera Dataset

We inferred a maximum-likelihood phylogenetic tree from the parsimony informative sites using IQ-TREE (*17*) to investigate the genetic relationship of the Malawi outbreak to that of other strains from around the world (sampled during 1957–2023; N = 2,778) (Appendix 1 Table 1). The reference set included 1,160 sequences from Africa, 898 from the Americas, 693 from Asia, 26 from Europe, and 1 from Oceania. We determined phylogenetic signal using a likelihood mapping test in IQ-TREE (*17*). We used Treetime (*18*) to obtain a maximum-likelihood tree scaled in time employing a standard mutation rate of 0.0179 substitutions/SNP site/year, as estimated by phylodynamic inference, after rerooting the tree by oldest tip.

### Phylodynamic Inference

We investigated the phylogenetic relationships of the 49 new clinical strains from Malawi with strains within a monophyletic multicountry clade containing 31 strains collected in 2022 from Pakistan, 49 publicly available strains from the same outbreak in Malawi (*19*), 114 strains collected from South Africa in 2023 (*20*), and 20 strains collected from Zimbabwe in 2023 (*21*) (Appendix 1 Table 2). We determined phylogenetic signal using the likelihood mapping test in IQ-TREE (*17*) and estimated temporal signal by plotting the root-to-tip divergence using TempEst (*22*). We used the Bayesian framework to infer a posterior distribution of trees and estimate the time of the most recent common ancestor (tMRCA) of the sampled sequences. We considered different molecular clock models (strict or uncorrelated relaxed molecular clock) and demographic priors (constant or Bayesian Skygrid) (*23*). We used BEASTX version 10.5.0-β5 (*24*) to run Markov chain Monte Carlo samplers for 500 million generations, sampling every 50,000 generations, which was sufficient to achieve mixing of the Markov chain as evaluated by effective sampling size >200 for all parameter estimates under a given model. We performed hypothesis testing for best molecular clock, demographic model, by obtaining marginal likelihood estimates via path sampling and stepping-stone methods for each model to be compared, then calculating the Bayes factor (BF). BF is the ratio of the of the null (*H_0_*) and the alternative hypothesis (*H_A_*) marginal likelihood estimation s (*25*), where *ln*BF<0 indicates support for *H_0_*; *ln*BF<2, negligible difference; 2<*ln*BF<6, strong support for *H_A_*; and *ln*BF>6, decisive support for *H_A_*^46^ (Appendix 2 Table 1). We estimated the mutation rate at 0.0179 substitutions/SNP site/year, which is in line with previous rates for cholera (*26*,*27*). We obtained the maximum clade credibility (MCC) tree from the posterior distribution of trees using optimal burn-in with TreeAnnotator (https://beast.community/programs). We manipulated the MCC phylogeny in R using the package ggtree as described (*28*) for publishing purposes. We inferred the geographic origin of the epidemic in Malawi using discrete trait model with asymmetric transition (migration) and Bayesian stochastic search variable selection, enforcing the best molecular clock and demographic model (uncorrelated relaxed molecular clock and Bayesian Skygrid) in BEASTX. BF values of 3–20 indicate positive evidence, values of 20–150 indicate strong evidence, and values >150 indicate very strong evidence (*29*,*30*) (Appendix 2 Table 2). We investigated cholera movement across Malawi, South Africa, and Zimbabwe by ancestral state reconstruction using continuous traits with the migration extension of TreeTime (*18*). We used a custom Python script to estimate dates of exchange events. We mapped results using the R packages maptools, raster, rgdal, and sf (The R Project for Statistical Computing, https://www.r-project.org). XML, MCC tree, ML phylogeny, and Treetime migration files are available at https://github.com/cmavian/cholera_Malawi_2022-2023.

We used the WebPlotDigitizer tool (https://apps.automeris.io/wpd) to extract data from a WHO report on cholera in the Africa region (https://iris.who.int/bitstream/handle/10665/366745/AFRO%20Cholera%20Bulletin.06.pdf). The report provided daily information on cholera cases and deaths within Malawi until April 4, 2023.

### Estimation of Time Varying Reproduction Number 

We inferred the instantaneous reproduction number over time (R_t_), at the national level, for Malawi using a semimechanistic Bayesian framework described in Bhatt et al. (*31*). To estimate the model, we used the epidemia package in R (https://imperialcollegelondon.github.io/epidemia), which enables us to estimate the effects of flooding and vaccinations as covariates. The estimate of R_t_ is based on daily national case counts, flooding, and vaccination data. We report the mean R_t_ estimate and 95% credible interval (CrI), a comparison of the observed and fitted case counts, and the effect sizes of our covariates (Appendix 2).

To check for robustness of our results, we estimated R_t_ with both flooding and vaccinations, only flooding, and with no covariates. We also confirmed that the estimation of R_t_ without any covariates is consistent with that of EpiEstim (*32*), which is an alternative implementation of a branching process model in R. Our R_t_ estimates were more stable because of the weekly random walk we used instead of daily data.

### Flooding Data and Processing

We obtained real-time or near–real-time flooding data by remotely sensed Earth observation imagery (*33*). Three sources are the Sentinels in Europe (*34*), the US National Aeronautics and Space Administration LANCE MODIS NRT global flood product (MCDWD) product using images generated from the Moderate Resolution Imaging Spectroradiometer instrument (https://www.earthdata.nasa.gov/global-flood-product), and Flood version 1.0 (*35*) using images generated from the Visible Infrared Imaging Radiometer Suite instrument available at the RealEarth website (https://floods.ssec.wisc.edu) and associated archives (https://jpssflood.gmu.edu). Sentinels in Europe are generally used to provide data for emergency response rather than long-term archives; MCDWD provided a long-term time series through 2022; and Flood provides ongoing daily and 5-day composites, which were downloaded for all of 2022 and then until April 13, 2023. Image values distinguish normal open water (value <99) from flood water, for which values >100 represent percentage flooding plus 100. The 5-day composite is the maximum value recorded during the period. The files are in simple tiled geotiff format; 2 tiles are required to cover the whole of Malawi, which must be combined and cropped before analysis.

### International Passenger Flight Data

We evaluated travel data generated from the International Air Transport Association (*36*) to quantify passenger volumes originating from international airports and arriving in Malawi. International Air Transport Association data account for ≈90% of passenger travel itineraries on commercial flights, excluding transportation via unscheduled charter flights; the remaining data are modeled using market intelligence.

## Results

### Overview of the Cholera Outbreak in Malawi

**Figure 1:**
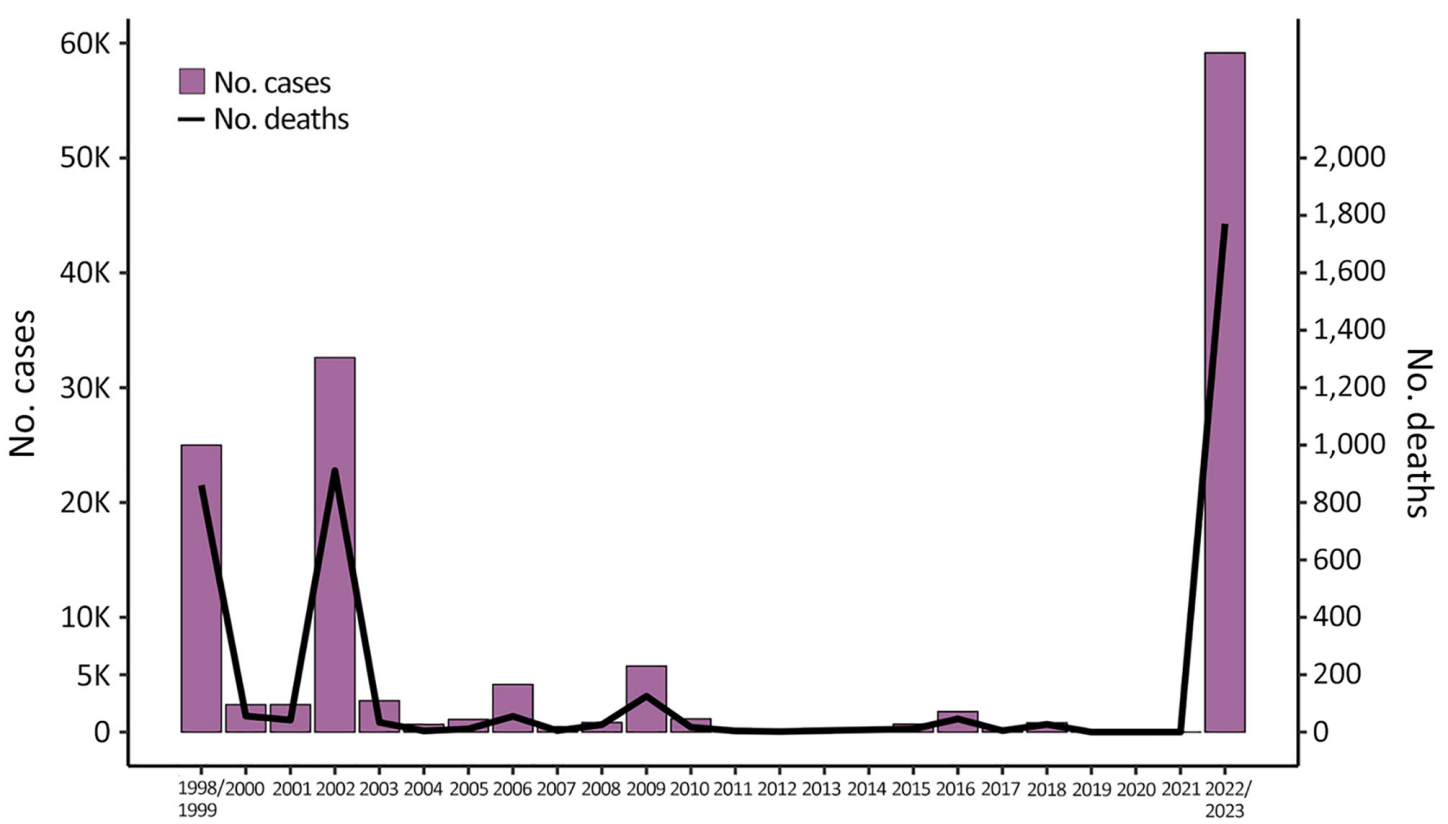
Cumulative case counts and deaths from cholera in Malawi by year, 1998–2023. Outbreaks occurred in 1998–1999, 2001–2002, 2008–2009, and 2022–2023. Scales for the y-axes differ substantially to underscore patterns but do not permit direct comparisons.

Before 2022, Malawi had experienced major cholera outbreaks in 1998–1999, 2001–2002, and 2008–2009 (*5*,*7*) (Figure 1). On March 3, 2022, Malawi declared a cholera outbreak after a confirmed case (symptom onset February 25, 2022) was found in Machinga District after Tropical Storm Ana (January 2022) and Cyclone Gombe (March 2022) caused severe flooding with resulting displacement of populations with low immunity to cholera and limited access to clean water and sanitation (*7*). Initially confined to flood-affected areas, the outbreak spread to central and northern regions by August 2022 (*7*). At the initiation of this study, 58,577 confirmed cases and 1,756 deaths were reported (Table; Figure 1). Given the consistent high case-fatality rate (>3%) and continued spread, the Malawi government declared a public health emergency on December 5, 2022. From December on, cases surged again affecting all regions, including Blantyre and Lilongwe, the country’s 2 largest cities (*7*). We obtained 70 *V. cholerae* isolates from stool samples collected during December 2022–March 2023. A total of 67 from the southern (n = 44), central (n = 18) and northern (n = 5) regions were processed into libraries (Table). From these 67 isolates, we generated 49 high-quality, near-complete *V. cholerae* genomes (Appendix 2 Figure 1).

**Table:** Epidemiologic characteristics of cholera outbreak by region, Malawi, 2022–2023

Region	No. samples sequenced	No. cases	No. deaths
Northern	5	4,373	94
Central	18	23,274	750
Southern	44	30,930	912

### Global Origin and Timing of 2022–2023 Cholera Outbreak 

**Figure 2:**
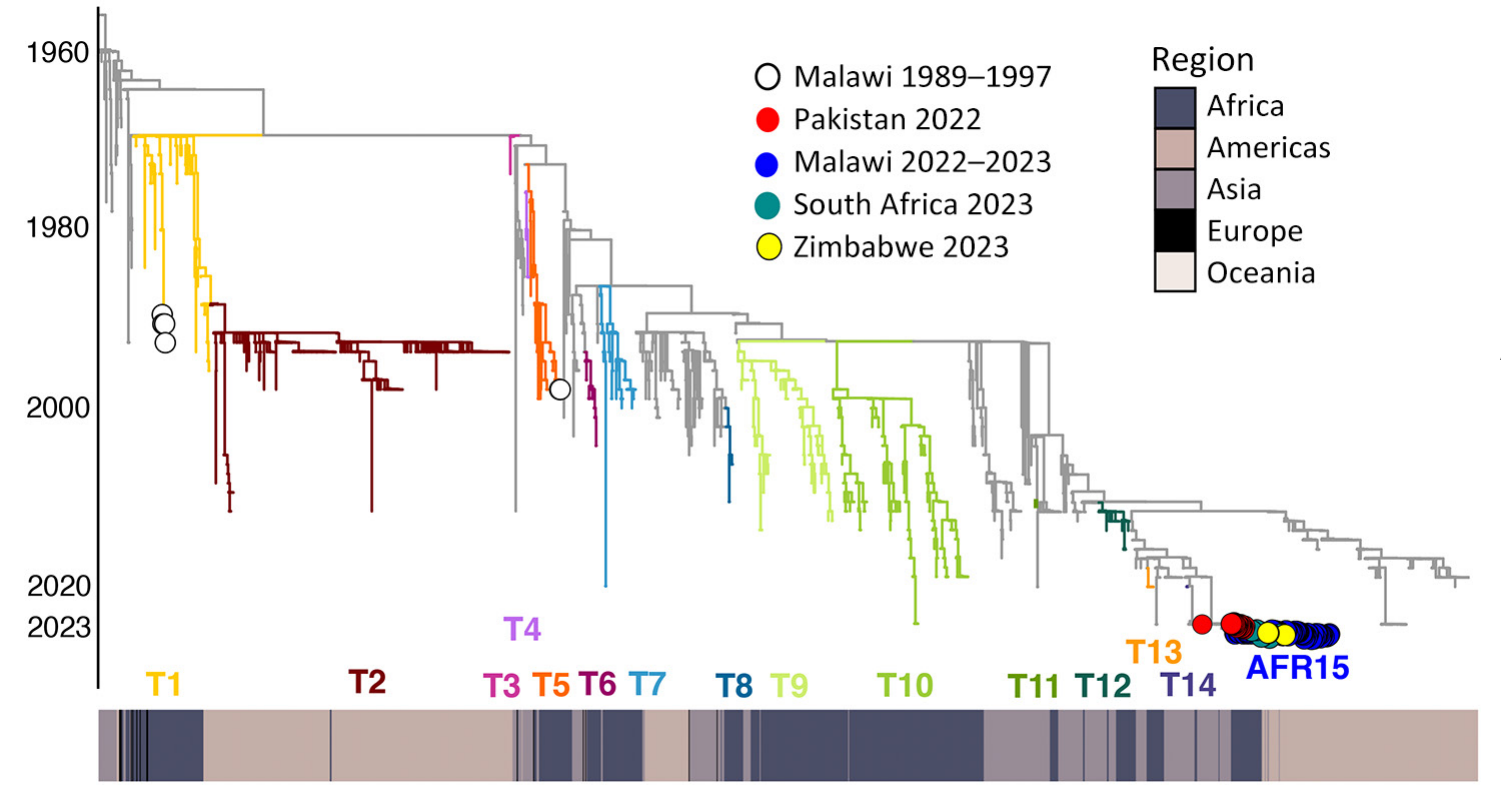
Phylogenetic history of cholera outbreaks within Malawi shown as part of a time-scaled maximum likelihood global phylogeny of 2,778 cholera genomes. Clade branches are colored by the previous 12 introduction events involving Africa (T1–T14 and AFR15) as described by Weill et al. (*9*). We denote the clade containing the genomes from the 2022–2023 outbreaks in Malawi, South Africa, and Zimbabwe as the AFR15 lineage. Heat map below the tree shows continent of sample origin.

**Figure 3:**
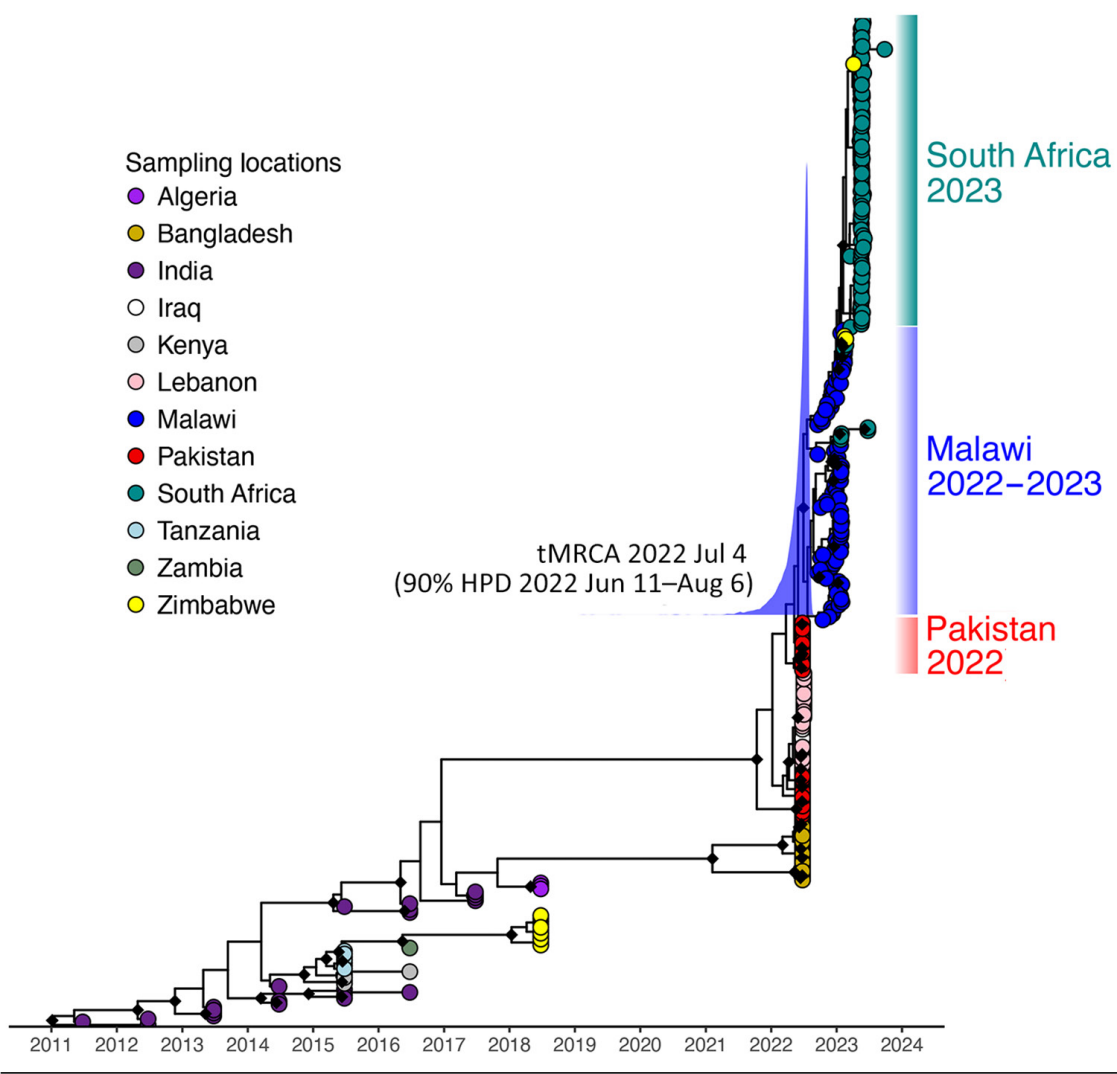
Maximum clade credibility phylogeny depicting the clade containing genomes from the 2022–2023 outbreak in Malawi (n = 89, sampled December 10, 2022–February 2023) with a basal clade of genomes sequenced from Pakistan in 2022. tMRCA estimate is shown. The blue curve shows posterior distribution of tMRCA. Black diamonds at nodes indicate posterior probability >0.9. HPD, highest posterior density; tMRCA, time to most recent common ancestor.

**Figure 4:**
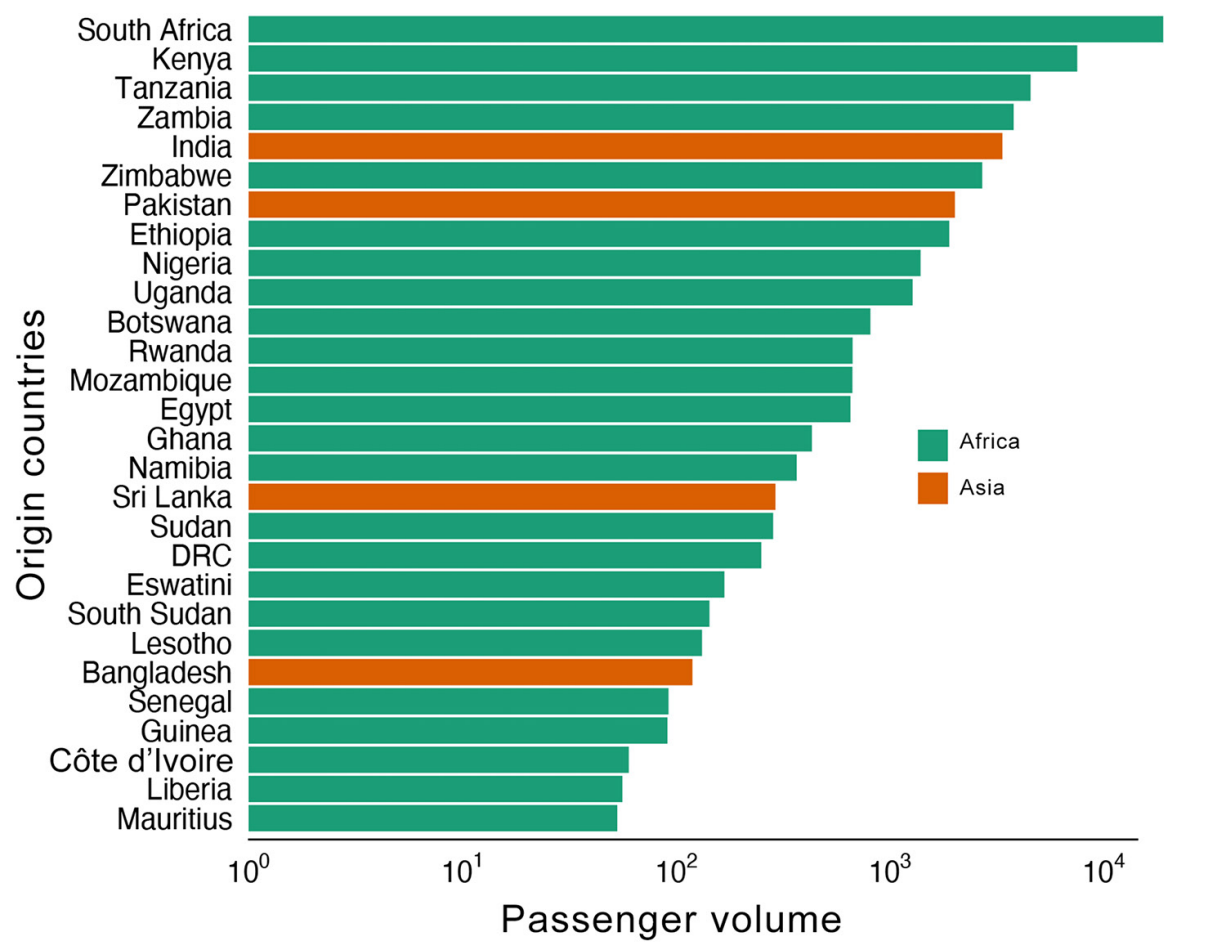
Number of passengers arriving by air travel into Malawi, by country of origin, January–June 2022. DRC, Democratic Republic of the Congo.

We constructed a time-scaled maximum-likelihood phylogeny based on high-quality SNPs of the 49 genomes from Malawi obtained in this study, 40 publicly available genomes (that passed quality filter) from the same outbreak (*19*), and 2,689 worldwide genomes (Figure 2). The genomes of the Malawi strains from this study clustered within a well-supported monophyletic clade (bootstrap >90%) together with the previously reported genomes from the same outbreak (*19*), denoting AFR15 introduction (*20*) (Figure 2). Those strains did not cluster with historical strains obtained from previous outbreaks within Malawi, which would indicate a new single introduction of cholera into the country. Instead, they were closely related to isolates from the 2022 outbreak in Pakistan (*37*) that clustered at the base of the clade, suggesting that the Malawi outbreak may have been caused by a strain introduction from Pakistan. However, we cannot reject that another unsampled country may have been involved in the origin of the Malawi cholera strain. The outbreak in Pakistan began in January 2022 and recorded >335,000 suspected cholera cases during January 15, 2022­‒March 15, 2023 (*38*). Phylogeographic analyses confirmed movement of cholera from Pakistan into Malawi (BF = 180.7) (Appendix 2 Table 3). The maximum clade credibility tree estimated tMRCA of the Malawi strains to be July 4, 2022 and determined a 95% highest posterior density (HPD) interval of June 11–August 6 (Figure 3). The tMRCA shared between Pakistan and Malawi outbreaks was June 4, 2022 (95% HPD interval of March 24–July 1, 2022), which suggests that the Malawi outbreak may have resulted from a long-range transmission event from Pakistan as early as March 2022, a close estimate to the beginning of the outbreak (*7*). Because the spread of cholera in Africa is associated with human movement (*9*), we queried the passenger volumes originating from international airports and arriving in Malawi (Figure 4). We found that Malawi was well connected to Pakistan by large numbers of air passengers traveling between them during the first half of 2022 (Figure 4).

### Cholera Transmission across Malawi and Africa

**Figure 5:**
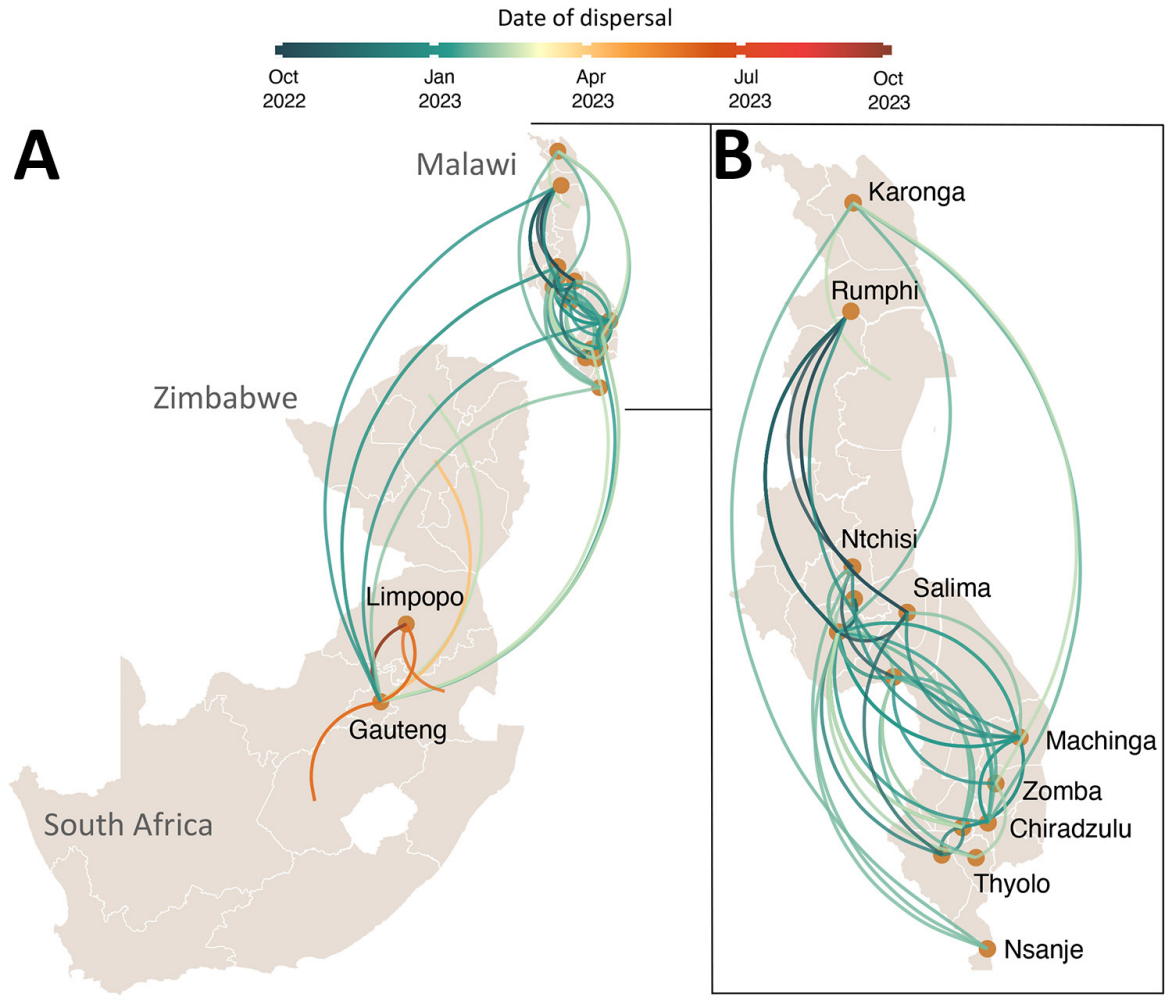
Spatiotemporal reconstruction of the spread of cholera in Malawi, South Africa, and Zimbabwe (A) and within Malawi (B) during the 2022–2023 outbreak. Circles represent nodes of the maximum likelihood phylogeny, and curved lines denote the links between nodes. Line colors indicates inferred time of occurrence. Directionality of spread is counterclockwise along each curve.

Next, we explored the spread of cholera within Malawi. Our phylogeographic analysis supported a single source introduction into Malawi with consequent spread within the country and confirmed epidemiologic reports indicating that the outbreak expanded from southern regions, initially affected by flooding, to the northern and central parts of the country (Figure 5). The analysis showed evidence of dispersal from multiple hubs, including Machinga Blantyre, and Lilongwe, in the epidemic acceleration phase (Figure 5). Introduction of cholera from Malawi into South Africa occurred through multiple transmission events (Figure 5). Phylogeographic analyses strongly suggested that cholera spread into South Africa from Malawi (BF = 50,222) on multiple separate introductions (Appendix 2 Table 3). The substantial flow of air passengers between Malawi and South Africa underscores the strong connectivity between the countries (Figure 4). After the introduction in South Africa, cholera futher spread into Zimbabwe from South Africa (BF = 25,109) on >2 separate occasions (Figure 5). Our analysis was limited by the sampling date range (September 2022–September 2023) and thus might not provide accurate insights into the dispersal patterns during the early phase of the outbreak.

### Effect of Severe Flooding in Malawi

**Figure 6:**
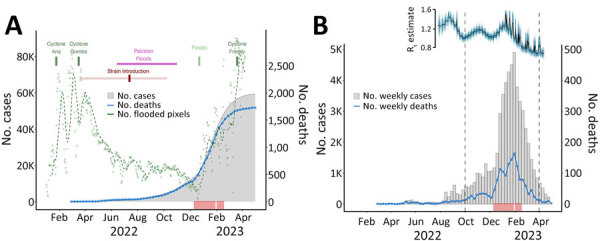
Relationships between confirmed cholera cases and cholera outbreak dynamics in Malawi during the 2022–2023 outbreak. A) Cyclones and flooding events affecting Malawi are shown. Green dots represent flooding conditions across Malawi as the daily number of pixels detected from remotely sensed satellite imagery; dashed green line indicates the moving average. Dark red mark indicates inferred time of introduction of a Pakistan strain into Malawi; the lighter shaded line indicates the 95% highest posterior density interval for the time estimate. B) Weekly number of newly confirmed cholera cases and deaths in Malawi during February 2022‒April 2023. Estimation of R_t_ and association with other variables is shown above the case data for September 2022‒April 2023 with plots of posterior distributions, median (black), and sample paths (blue). In both plots, red rug plot below charts indicates temporal distribution of cases sequenced in our study. R_t_, time-varying reproduction number.

Because the 2022 Pakistan cholera outbreak was exacerbated by major floods during June‒October 2022 (*39*), we next examined the Malawi outbreak in light of the extreme weather events that occurred in 2022 (Figure 6, panel A; Appendix 2 Figure 2). The outbreak started after Tropical Storm Ana (January 28, 2022), which caused widespread flooding and damage in southern Malawi and left >200,000 persons displaced, many in emergency camps without adequate access to safe water and sanitation (*7*). Soon after the start of the outbreak, Cyclone Gombe caused further flooding (as measured by area flooded) and heavy damage across southern Malawi, including many areas already affected by Tropical Storm Ana (Appendix 2 Figure 2). The interval of the introduction of cholera into Malawi (95% HPD March 2022–August 2022) correlates with the beginning of the reported cases for the current cholera outbreak in the country (Figure 6, panel A; Appendix 2 Figure 2) and overlaps with flooding in Pakistan that exacerbated the ongoing cholera outbreak in that country. Initial floods in Malawi did not seem to correlate with amplification of cases, suggesting that the introduction was followed only by low circulation restricted to the flood-affected areas in the southern region (Appendix 2 Figure 2). Floods that occurred during the normal rainy season in late November 2022 likely intensified transmission; cases surged particularly in Blantyre and Lilongwe, the 2 main urban centers (Appendix 2 Figure 2) (*7*). The rapid surge of cases was likely spurred on by the extreme flooding events causing a lack of access to safe drinking water, poor sanitation and hygiene, and displacement of a vulnerable population (Figure 6) (*7*). Because we did not have genomes from the early phase of the outbreak in our study and because the introduction interval was relatively wide, spanning several months, we must consider an alternative hypothesis: the initial floods may have led to low-level circulation of endemic cholera strains, initiating the outbreak in Malawi. Subsequently, during the dry season, the introduction of the strain from Pakistan occurred with minimal transmission until later floods permitted the amplified transmission of a potentially more transmissible strain.

We estimated that R_t_ from daily case data increased after November 28, 2022, to a maximum of 1.619 on December 29, 2022, before starting to decline in early January 2023; it was consistently <1 from mid-February 2023 and at 0.704 at the end of the study period (Figure 6, panel B). Flooding (Figure 6, panel A) was positively associated with R_t_ (0.262 [95% CrI 0.003–0.509]) (Appendix 2 Figure 3, panel D). 

Two oral cholera vaccination campaigns have been conducted since the onset of the outbreak; a total of 2,825,229 doses were administered by December 2, 2022, covering 96.8% of the population residing in communities with high risk and burden of cholera (*7*). We estimated a negative association between the vaccination campaigns and R_t_ (−0.320 [90% CrI −0.638 to −0.013]) (Appendix 2 Figure 3, panel D) but note that the association is only significant at a 90% CrI. Our results were robust to a varying level of the initial susceptible population (S_0_), which is important because the initial vaccination campaign in early 2022 may have reduced the S_0_ further than the S_0_ we used as our baseline, and we do not know about any prior immunity in the community caused by previous outbreaks (Appendix 2 Figure 3).

## Discussion

In this genomic analysis, we provide evidence of a link between the recent large cholera outbreaks in Pakistan and Malawi, consistent with previous genomic analyses revealing the importance of long-range *V. cholerae* transmission events between Asia and Africa (*8*,*9*). However, we cannot rule out that another unsampled country could be the origin of both the Malawi and Pakistan outbreaks. Our main findings support another recent analysis of the Malawi outbreak (*19*), which was linked to the strains previously identified in Asia, and another report of cases from South Africa in 2023 epidemiologically linked to Malawi (*20*). The extreme weather events and humanitarian crises in Malawi provided a suitable environment for the amplified spread of *V. cholerae*, and the subsequent movement of large numbers of persons may have enabled its spread to susceptible populations in areas relatively unaffected by cholera for more than a decade.

The relatively narrow sampling date range for this genomic analysis meant that we cannot confidently differentiate whether the introduction of *V. cholerae* was responsible for initiating the outbreak or if it only contributed to the later expansion of the outbreak through multiple transmission chains. Further sequencing of isolates from earlier stages of the outbreak could elucidate the initial dynamics, emphasizing the criticality of controlling cholera outbreaks and preventing introductions during the dry season to prevent escalation during subsequent flooding seasons. We are also working with partners in other heavily affected countries in Africa to conduct genomic sequencing of *V. cholerae* isolates, which will help with understanding the extent of cross-border regional transmission at different phases of the outbreak.

We modeled R_t_ over the course of the outbreak, incorporating satellite data to assess the effect of flooding on cholera transmission, which is particularly important in studying a waterborne disease like cholera. A substantial positive association between flooding and reproduction numbers explained the sustained high case numbers in January. The decline after February 2023 was likely caused by a successful vaccination campaign, infection-acquired immunity, and depletion of susceptible persons. Our model, based on national case data, lacked precise vaccination rollout details, which limited its accuracy. Future research should use spatially disaggregated data to better assess the effects of flooding and vaccination on cholera dynamics. Overall, our study highlights the need for coordinated global and regional cholera prevention and control efforts and the importance of heightened awareness, data sharing, and preparedness whenever outbreaks occur in any part of the world (*40*). 

Appendix 1 Cholera strains used in study of genomic surveillance of a climate-amplified cholera outbreak in Malawi, 2022–2023.

Appendix 2 Additional information about genomic surveillance of a climate-amplified cholera outbreak, Malawi, 2022–2023.
