# Supplementary material for: Genomic Surveillance of Climate-Amplified Cholera Outbreak, Malawi, 2022–2023
Source: Emerg Infect Dis. 2025 Jun;31(6):1090–9. doi: 10.3201/eid3106.240930 (PMC12123921; doi:10.3201/eid3106.240930)
Supplement: Appendix 2 — Additional information about genomic surveillance of a climate-amplified cholera outbreak, Malawi, 2022–2023. [file 24-0930-Techapp-s2.pdf]

Article DOI: https://doi.org/10.3201/eid3106.240930

*EID cannot ensure accessibility for supplementary materials supplied by authors. Readers who have difficulty accessing supplementary content should contact the authors for assistance.*

# Genomic Surveillance of Climate-Amplified Cholera Outbreak, Malawi, 2022–2023

## Appendix 2

## Supplemental Material and Methods

***Methods:*** **Estimation of time-varying reproduction number ($R_t$) and association with other variables**

We model the number of new cases, $i_t$', at time t>0, through a renewal equation, and these new cases are moderated by $R_t$ (Bhatt et al. (*1*)).

$$i_t' = R_t \cdot \sum_{s<t} i_s g_{t-s}$$

$$i_t = S_{t-1} \cdot \left(1 - \exp\left(-\frac{i_t'}{P}\right)\right)$$

where g is approximated by the serial interval, that is the distribution of time from symptom onset of the primary case to the symptom onset of the secondary case. For cholera we assume a mean serial interval of 5 days, a standard deviation of 8 days and that g is gamma distributed with a 1-day shift (*2*). $S_t$ is the size of the susceptible population at time t and P is the size of the population and the second equation ensures that $i_t$' accounts for the depletion of susceptible individuals in the population. We give the initial susceptible population $S_0$ a N(0.95,0.1) prior as this provides a conservative prior which assumes that a very large majority of the population is susceptible.

We model case observations as $o_t = \sum_{s<t} i_t' \cdot \pi_{t-s}$ where π is the infection to onset distribution with a median of 1.4 days (*3*).

We can form a linear predictor, consisting of fixed effects and autocorrelation terms, which is then transformed via a link function, to model the reproduction number.

$$R_t = g^{-1}\left(\beta_0 + \sum_k X_{t,k}\beta_k + \epsilon_{w(t)}\right)$$

$$R_t = R_0 \cdot g^{-1}\left(\sum_k X_{t,k}\beta_k + \epsilon_{w(t)}\right)$$

We use a *log*-link function as this ensures nonnegative reproduction numbers which don't grow exponentially. We use $R_0 \sim \text{Normal}(1.56, 0.2)$ as a prior, where the mean is consistent with Mukandavire et al. (*4*).

$\epsilon_{w(t)}$ is a weekly random walk (we use a weekly random walk rather than a daily random walk to obtain more stable $R_t$ estimates), $X_{t,k}$ are covariates of interest and $\beta_k$ their respective effect sizes.

We use 'epidemia' to obtain the posterior distribution with priors as provided above.

We use two covariates in our analysis:

1. *Flooding data (see details in Methods section of the main text)* – we transform available flooding data as follows
    1. 1-day difference in flooded area
    2. An indicator if the difference is bigger than 1,000 pixels
    3. Delay by 4 days to account for mean serial interval

This transformation creates an indicator time series of significant rainfall and shifts it to account for the mean serial interval. Similar to Camacho et al. (*2*), it is a mechanistic representation of rainfall's impact.

2. *Vaccinations* – two vaccination campaigns took place in Malawi against cholera (WHO) (*5*). The first vaccination campaign is not modeled as it was early in 2022. The second vaccination campaign is modeled such that we assume that the roll-out takes place over 3 months, with a delay of 10 days to account for the time lag until the vaccine is effective. We only estimate the effect size of vaccination as an intervention.

The results of the model are provided (Appendix 2 Figure 3).

We conducted sensitivity analysis with respect to our prior $S_0$, considering a 95%, 75%, and 40% susceptible population, and found that the impact on the effect sizes for rainfall and vaccination was minimal (Appendix 2 Figure 3).

### References


1. Bhatt S, Ferguson N, Flaxman S, Gandy A, Mishra S, Scott JA. Semi-mechanistic Bayesian modelling of COVID-19 with renewal processes. J R Stat Soc Ser A Stat Soc. 2023;186:601–15. https://doi.org/10.1093/jrsssa/qnad030

2. Camacho A, Bouhenia M, Alyusfi R, Alkohlani A, Naji MAM, de Radiguès X, et al. Cholera epidemic in Yemen, 2016–18: an analysis of surveillance data. Lancet Glob Health. 2018;6:e680–90. PubMed https://doi.org/10.1016/S2214-109X(18)30230-4

3. Azman AS, Rudolph KE, Cummings DAT, Lessler J. The incubation period of cholera: a systematic review. J Infect. 2013;66:432–8. PubMed https://doi.org/10.1016/j.jinf.2012.11.013

4. Mukandavire Z, Morris JG Jr. Modeling the epidemiology of cholera to prevent disease transmission in developing countries. Microbiol Spectr. 2015;3:3.3.21. PubMed https://doi.org/10.1128/microbiolspec.VE-0011-2014

5. Malawi WHO. Vaccines help battle cholera outbreak in Malawi. 2023. https://www.afro.who.int/countries/malawi/news/vaccines-help-battle-cholera-outbreak-malawi

**Appendix 2 Table 1.** Model selection of molecular clock and Bayesian demographic models to infer time-structured phylogeny.

| Model | $ln(ML)_{SS}$ | $Ln(BF)_{SS}$ | $ln(ML)_{PS}$ | $Ln(BF)_{PS}$ |
|---|---|---|---|---|
| SC CONST | -2239.2 | 12.7 | -2238.3 | 12.5 |
| RC CONST | -2226.5 | 12.7 | -2225.8 | 12.5 |
| RC CONST | -2226.5 | 0.5 | -2225.8 | 0.2 |
| RC BSG | -2226 | 0.5 | -2225.6 | 0.2 |

*Log of marginal likelihood estimate, ln (MLE) values obtained by Stepping Stone (SS) and Path Sampling (PS), are reported for models that either used as priors: strict (SC) or uncorrelated relaxed lognormal (UCLN) molecular clocks, and constant (CONST), non-parametric Bayesian skygrid (BSG) demographic models.

**Appendix 2 Table 2.** Bayes factor values and directionality of inferred migration rates*

| Bayes factor | From | To |
|---|---|---|
| 14.6 | Algeria | Bangladesh |
| 0.8 | Algeria | India |
| 0.8 | Algeria | Iraq |
| 1.5 | Algeria | Kenya |
| 0.9 | Algeria | Lebanon |
| 0.9 | Algeria | Malawi |
| 3.8 | Algeria | Pakistan |
| 0.8 | Algeria | South_Africa |
| 1.3 | Algeria | Tanzania |
| 0.9 | Algeria | Zambia |
| 0.9 | Algeria | Zimbabwe |
| 2.0 | Bangladesh | Algeria |
| 1.0 | Bangladesh | India |
| 0.9 | Bangladesh | Iraq |
| 1.1 | Bangladesh | Kenya |
| 1.0 | Bangladesh | Lebanon |
| 1.0 | Bangladesh | Malawi |
| 2.7 | Bangladesh | Pakistan |
| 0.9 | Bangladesh | South_Africa |
| 1.0 | Bangladesh | Tanzania |
| 0.9 | Bangladesh | Zambia |
| 0.9 | Bangladesh | Zimbabwe |
| 55.3 | India | Algeria |
| 4.0 | India | Bangladesh |
| 0.5 | India | Iraq |
| 15.5 | India | Kenya |
| 0.5 | India | Lebanon |
| 0.4 | India | Malawi |
| 7.6 | India | Pakistan |
| 0.5 | India | South_Africa |
| 5.1 | India | Tanzania |
| 0.5 | India | Zambia |
| 0.6 | India | Zimbabwe |
| 1.8 | Iraq | Algeria |
| 1.2 | Iraq | Bangladesh |
| 1.6 | Iraq | India |
| 1.2 | Iraq | Kenya |
| 1.4 | Iraq | Lebanon |
| 1.1 | Iraq | Malawi |
| 1.4 | Iraq | Pakistan |
| 1.1 | Iraq | South_Africa |
| 1.2 | Iraq | Tanzania |
| 1.1 | Iraq | Zambia |
| 1.1 | Iraq | Zimbabwe |
| 1.7 | Kenya | Algeria |
| 1.4 | Kenya | Bangladesh |
| 1.0 | Kenya | India |
| 1.0 | Kenya | Iraq |
| 0.8 | Kenya | Lebanon |
| 0.8 | Kenya | Malawi |
| 2.1 | Kenya | Pakistan |
| 0.8 | Kenya | South_Africa |
| 20.0 | Kenya | Tanzania |
| 1.1 | Kenya | Zambia |
| 1.0 | Kenya | Zimbabwe |
| 1.0 | Lebanon | Algeria |

| Bayes factor | From | To |
|---|---|---|
| 1.0 | Lebanon | Bangladesh |
| 0.9 | Lebanon | India |
| 169.8 | Lebanon | Iraq |
| 0.8 | Lebanon | Kenya |
| 0.9 | Lebanon | Malawi |
| 1.1 | Lebanon | Pakistan |
| 0.7 | Lebanon | South_Africa |
| 0.7 | Lebanon | Tanzania |
| 0.7 | Lebanon | Zambia |
| 0.8 | Lebanon | Zimbabwe |
| 0.7 | Malawi | Algeria |
| 0.5 | Malawi | Bangladesh |
| 0.6 | Malawi | India |
| 0.4 | Malawi | Iraq |
| 0.6 | Malawi | Kenya |
| 0.5 | Malawi | Lebanon |
| 0.7 | Malawi | Pakistan |
| 50229.3 | Malawi | South_Africa |
| 0.5 | Malawi | Tanzania |
| 0.5 | Malawi | Zambia |
| 0.5 | Malawi | Zimbabwe |
| 1.0 | Pakistan | Algeria |
| 1.2 | Pakistan | Bangladesh |
| 0.7 | Pakistan | India |
| 1.3 | Pakistan | Iraq |
| 0.8 | Pakistan | Kenya |
| 158.3 | Pakistan | Lebanon |
| 180.7 | Pakistan | Malawi |
| 0.6 | Pakistan | South_Africa |
| 0.7 | Pakistan | Tanzania |
| 0.6 | Pakistan | Zambia |
| 0.6 | Pakistan | Zimbabwe |
| 0.9 | South_Africa | Algeria |
| 0.6 | South_Africa | Bangladesh |
| 0.8 | South_Africa | India |
| 0.6 | South_Africa | Iraq |
| 0.6 | South_Africa | Kenya |
| 0.6 | South_Africa | Lebanon |
| 0.6 | South_Africa | Malawi |
| 0.7 | South_Africa | Pakistan |
| 0.7 | South_Africa | Tanzania |
| 0.6 | South_Africa | Zambia |
| 25109.5 | South_Africa | Zimbabwe |
| 1.3 | Tanzania | Algeria |
| 1.1 | Tanzania | Bangladesh |
| 0.9 | Tanzania | India |
| 0.8 | Tanzania | Iraq |
| 5.5 | Tanzania | Kenya |
| 0.8 | Tanzania | Lebanon |
| 0.6 | Tanzania | Malawi |
| 1.4 | Tanzania | Pakistan |
| 0.9 | Tanzania | South_Africa |
| 110.5 | Tanzania | Zambia |
| 1.9 | Tanzania | Zimbabwe |
| 1.2 | Zambia | Algeria |
| 0.9 | Zambia | Bangladesh |
| 1.1 | Zambia | India |
| 0.8 | Zambia | Iraq |
| 0.9 | Zambia | Kenya |
| 0.9 | Zambia | Lebanon |
| 0.9 | Zambia | Malawi |
| 1.0 | Zambia | Pakistan |
| 1.7 | Zambia | South_Africa |
| 0.8 | Zambia | Tanzania |
| 38.3 | Zambia | Zimbabwe |
| 2.4 | Zimbabwe | Algeria |
| 1.6 | Zimbabwe | Bangladesh |
| 2.5 | Zimbabwe | India |
| 1.2 | Zimbabwe | Iraq |

| Bayes factor | From | To |
|---|---|---|
| 1.3 | Zimbabwe | Kenya |
| 1.3 | Zimbabwe | Lebanon |
| 1.1 | Zimbabwe | Malawi |
| 1.7 | Zimbabwe | Pakistan |
| 1.0 | Zimbabwe | South_Africa |
| 1.4 | Zimbabwe | Tanzania |
| 1.7 | Zimbabwe | Zambia |

* Bayes Factor [BF] values of 3–20 indicate positive evidence (highlighted in yellow), BF of 20–150 indicate strong (highlighted in orange), and values greater than 150 indicate very strong evidence (highlighted in green).

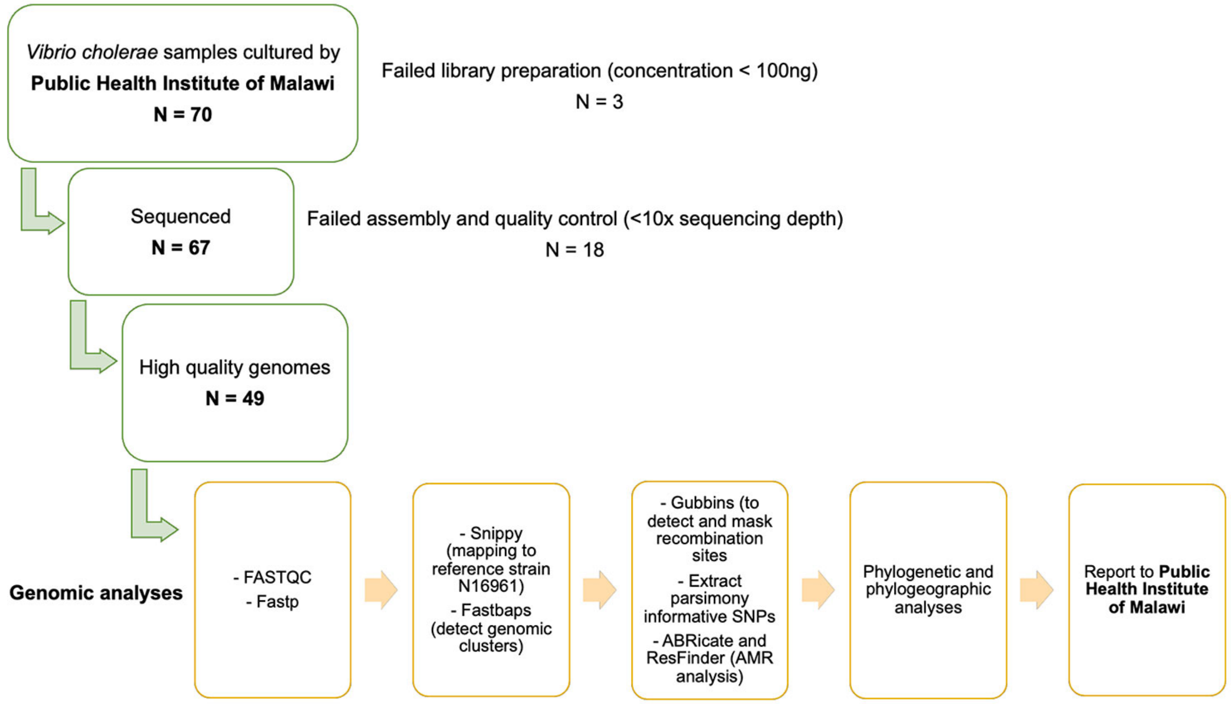


**Appendix 2 Figure 1.** Cholera sequencing workflow. Out of 70 *Vibrio cholerae* samples cultured by the Public Health Institute of Malawi, three samples failed library preparation as DNA concentration was less than 100ng. Out of 67 samples successfully sequenced, 18 samples failed assembly and quality control as sequencing depth was less than 10X. We obtained 49 high-quality genomes. The genomic analyses were as follow: raw data was processed with fastp and FASTQC, Snippy was used for reference assembly, Fastbaps for cluster detection, Gubbins was used for detect and mask recombination sites, MEGAX was used to extract parsimony informative SNPs, ABRicate and ResFinder for AMR analsyis. SNPs were used to infer phylogenetic trees and for phylogegraphic analyses. The data was reported to the Public Health Institute of Malawi.

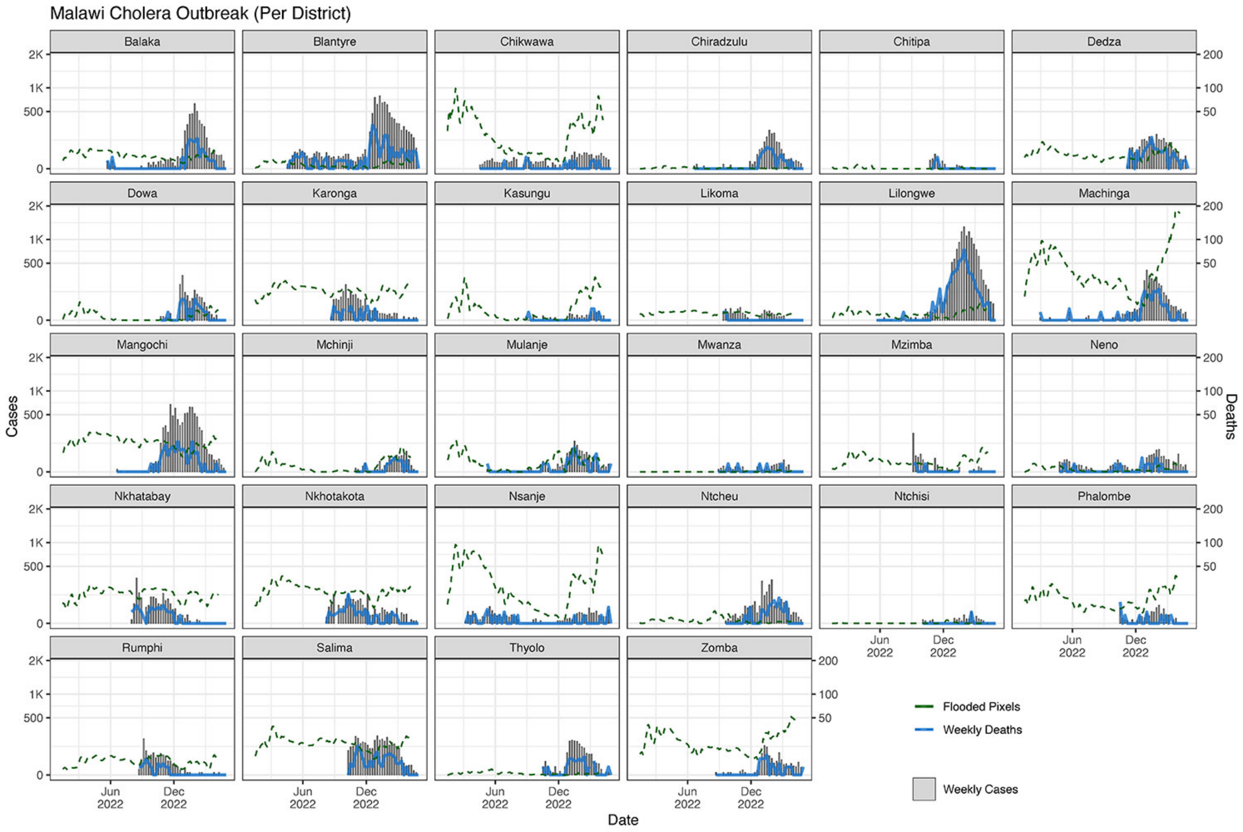


**Appendix 2 Figure 2.** Weekly number of confirmed cholera cases and deaths per district in Malawi are depicted on the y-axes from February 2022 to April 2023**.** Green dots portray the daily number of pixels demonstrating flooding conditions across districts of Malawi as detected from remotely sensed satellite imagery with the moving average shown as the dashed green line.

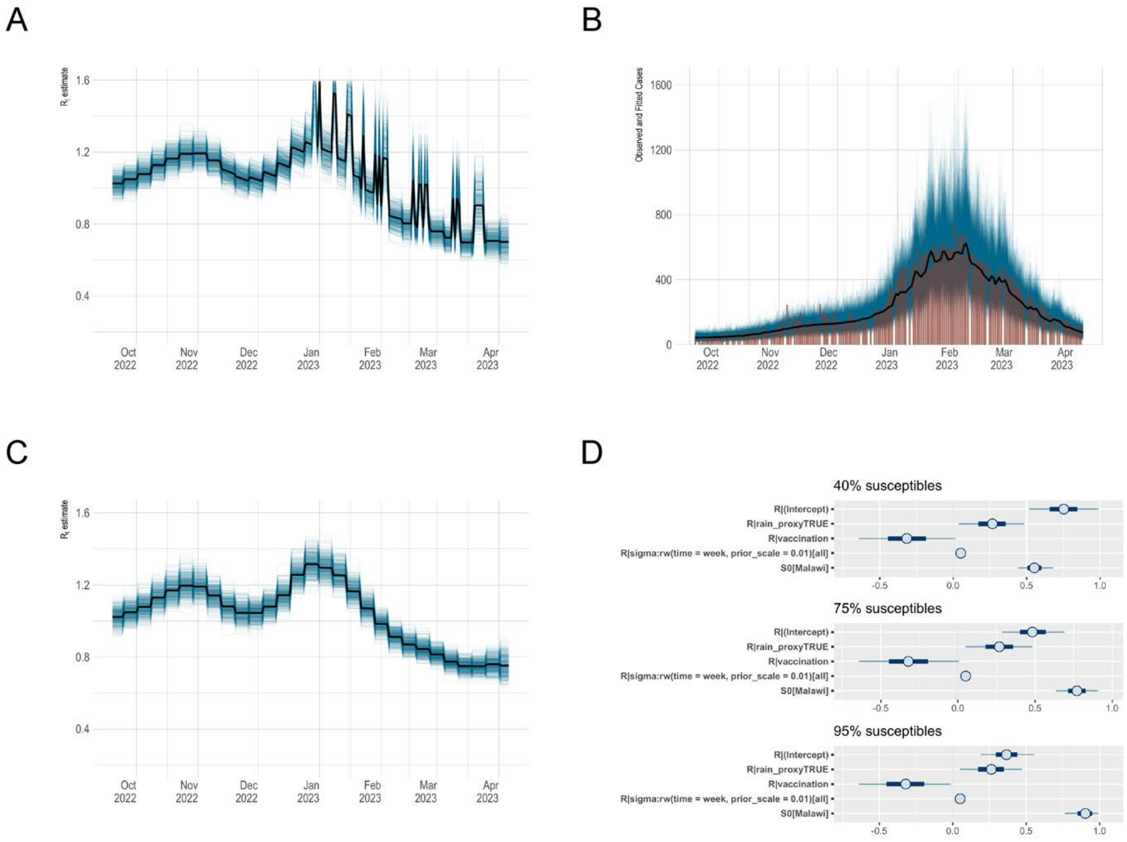


**Appendix 2 Figure 3.** Estimation of time-varying reproduction number ($R_t$) and association with other variables. Plots of posterior distributions, median (black) and sample paths (blue). (A) Estimated $R_t$ for model including flooding and vaccination. (B) Observed cases (brown bars) and sample paths. (C) Estimated $R_t$ for model without covariates. (D) Effect sizes for covariates for different priors of $S_0$ (as outlined in section S1) with 50% (bold) and 90% (thin) posterior interval estimates.
